# The Relationship Between Enlarged Perivascular Spaces and Cognitive Function: A Meta-Analysis of Observational Studies

**DOI:** 10.3389/fphar.2020.00715

**Published:** 2020-05-15

**Authors:** Wanxin Jie, Guanghong Lin, Zhou Liu, Haihong Zhou, Lifeng Lin, Guocong Liang, Mingqian Ou, Meijun Lin

**Affiliations:** ^1^Department of Neurology, Institute of Neurology, Guangdong Key Laboratory of Age-Related Cardiac and Cerebral Diseases, Affiliated Hospital of Guangdong Medical University, Zhanjiang, China; ^2^Medical Intensive Care Unit, Central People’s Hospital of Zhanjiang, Zhanjiang, China

**Keywords:** enlarged perivascular spaces (ePVS), impaired cognition, meta-analysis, cohort studies, odds ratio (OR)

## Abstract

Enlarged perivascular spaces (ePVS), visible on magnetic resonance imaging (MRI), are associated with aortic pulse wave changes produced by arterial stiffening. However, the relationship between ePVS and cognition is still unclear. We aimed to benchmark current knowledge of associations between ePVS and cognitive function using a meta-analysis of all available published data. We searched three databases for studies examining ePVS and cognition, identified seven studies involving 7,816 participants, plotted multivariate-adjusted odds ratio (OR) and 95% CI and generated summary OR with a fixed effects model. EPVS were related to the risk of impaired cognition (OR = 1.387, 95% CI = 1.198–1.606, z=4.38, P<0.001) with low heterogeneity. There was publication bias, which could be corrected by trimming and supplementation (OR=1.297, 95% CI= 1.130–1.490). EPVS were associated with impaired cognition and may be a sign of cognitive impairment rather than particular diseases. More studies are required to validate ePVS as a measurable risk marker for cognition using consistent methods to determinea characteristic appearance of ePVS.

## Introduction

Perivascular spaces (PVS), also known as Virchow-Robin spaces, are a fluid-filled structure lined with tubes found at specific locations throughout the brain (Rudie et al., 2018). They can be seen on magnetic resonance imaging (MRI) in the centrum semiovale (CSO), basal ganglia (BG), the hippocampus (HP) and white matter (WM) (Schwartz et al., 2019). With increasing age, arteries become stiffer and reflected pressure waves return to the central aorta earlier in the cardiac cycle, producing augmentation of the systolic pulse wave and central arterial systolic pressure. It has been suggested that the development of cerebral small vessel disease (SVD) might represent a downstream effect of this haemodynamic change (Elias et al., 2009). In addition, recent findings have supported the hypothesis that changes in aortic pulse waves produced by arterial stiffening were associated with the presence of enlarged PVS (ePVS) (Thomas et al., 2019). An increasing number of reports over the last two decades have detailed associations of ePVS on MRI, as a marker of SVD, with cognitive function (Francis, 2019). However, the relationship between ePVS and cognition is still unclear. Some articles have suggested that ePVS are related to cognition, while others have shown that they are not.

We aimed to benchmark current knowledge of the associations between ePVS and cognitive function, using meta-analysis of all available published data.

## Methods

We used the meta-analysis of observational studies in epidemiology (MOOSE) (Stroup et al., 2000) checklist.

### Evaluation Procedure

Two independent investigators (WJ and GHL) selected all relevant studies based on title and abstract, retrieved selected full texts, performed eligibility assessments, extracted data, and assessed risk of bias. Disagreements between the reviewers were resolved by consensus. A third independent reviewer (ZL) resolved any persisting disagreements.

### Information Sources and Search Strategy

We comprehensively searched for studies published in full up to 27 August 2019, in PubMed, MEDLINE, and Ovid EMBASE. The following search terms were used:’(((((((Virchow-Robin Spaces) OR Perivascular Spaces) OR Enlarged Virchow-Robin Spaces) OR Enlarged Perivascular Spaces) OR Virchow-Robin Spaces Enlargement) OR Perivascular Spaces Enlargement)) AND (((((((Cognitive) OR Cognition) OR Cognitive Disorder) OR Cognitive Impairment) OR Cognitive Dysfunction) OR Cognitive Impaired) OR Dementia)’. We applied no language or calendar date restrictions. We checked reference lists of eligible papers and hand searched related literature to identify further relevant studies.

### Study Selection

We aimed to include all papers that reported on ePVS associations with cognitive function. We included cohort studies in adults with standardized risk estimates that were reported as odds ratios (ORs), relative risks (RRs), or hazard ratios (HRs) and excluded case reports, animal studies and reviews without original data. Studies were also excluded if they reported on linear associations (expressed as β-coefficients) on the relation between ePVS and cognition.

### Data Extraction

We removed repetitive articles, screened the remaining titles and abstracts, removed irrelevant papers and assessed the remaining potentially eligible papers for inclusion. The following data were extracted: name of the first author, publication year, country of publication, publication type, sample source, sample size, age (mean), subject characteristics, follow-up time, grouping, ePVS location (BG, CSO, WM, or HP), the results of associations (ORs, RRs, or HRs with corresponding 95% CIs), adjustment factors, statistical methods, rating scale for ePVS, type of scanner and MRI sequence, and psychological assessment method. For those studies with many sets of adjustment factors, we extracted the most-adjusted risk estimate, and for those with groups, we merged their ORs.

### Quality Assessment

We evaluated the study quality with the Newcastle-Ottawa scale (NOS). The scale uses a “star” rating system (maximum of nine stars) to assess the quality of case-control and cohort studies based on three aspects: selection of participants, comparability of study groups, and ascertainment of outcomes of interest (Stang, 2010). If the study scored nine stars, it was considered to be of high quality. Studies with a score of seven or eight stars were considered to be of medium quality. However, if a study scored less than seven stars, it was considered to be of low quality.

### Statistical Analysis

We used a fixed effects model, plotted multivariate-adjusted ORs and 95% CI and generated summary ORs. Chi-square test was used to assess interstudy heterogeneity, and P <0.05 was considered statistically significant. Higgin’s I^2^ test was used to calculate the percentage of variance between studies due to heterogeneity rather than random factors. I^2^ of 25% or less was considered to be low, 26% to 50% or less moderate, 51%–75% or less high, and 76% or more was considered to be very high heterogeneity (Higgins and Thompson, 2002). The significance of the summary ORs was assessed using the Z-test, and a *P*-value < 0.05 was considered statistically significant. A sensitivity analysis was conducted to evaluate the stability of the results by systematically excluding a single study in each analysis. Funnel plots and Egger’s tests were used to investigate the potential publication bias. For the potential publication bias, we conducted a trim and fill analysis to yield an effect adjusted for funnel plot asymmetry. All statistical analyses were conducted using Stata software version 12.0(StataCorp).

## Results

### Retrieved Studies and Characteristics

**Figure 1:**
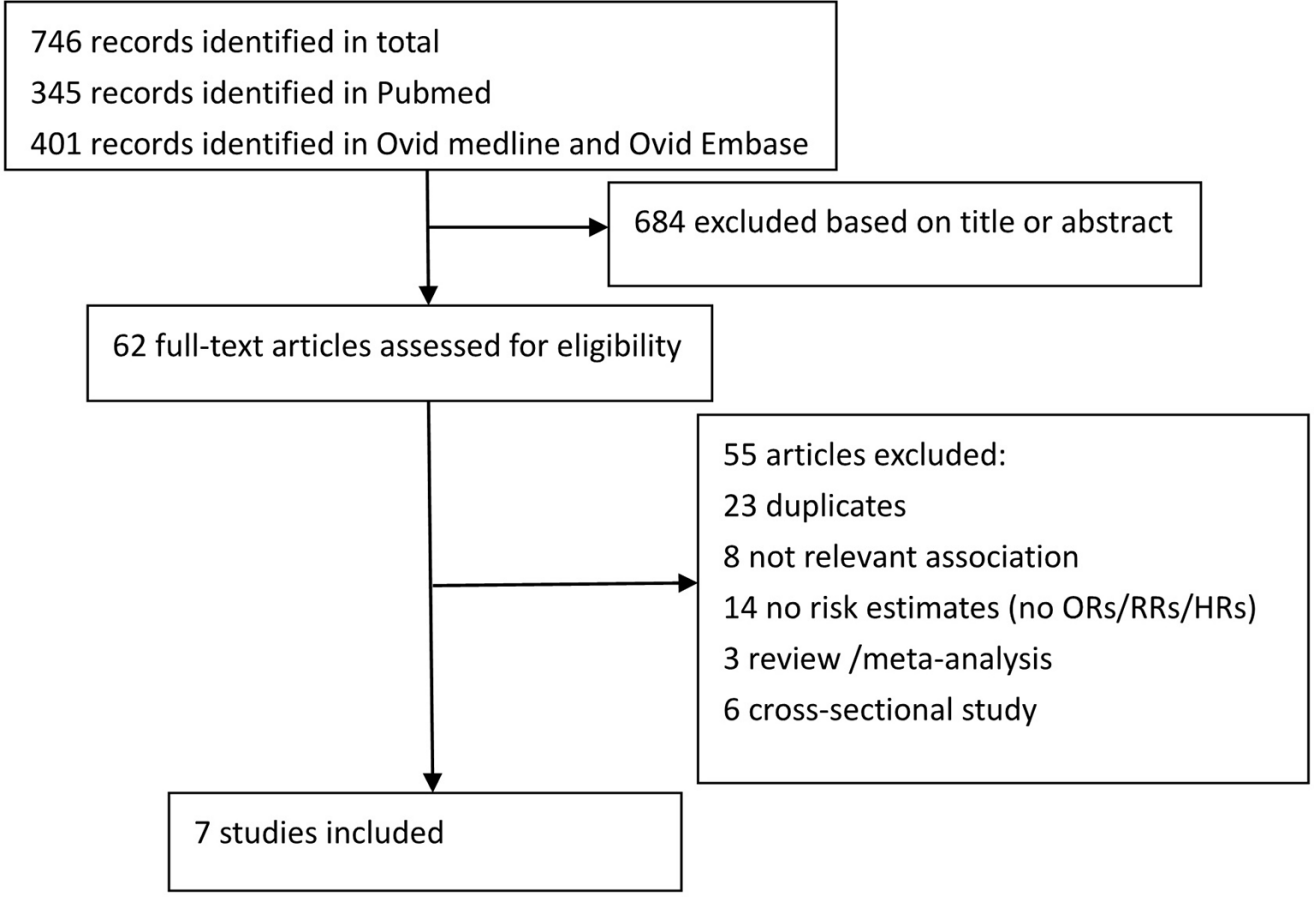
Flow chart of study selection.

We identified 746 articles. After screening titles and abstracts and deleting duplicate papers, we obtained 39 study reports for full-text review. After a full-text review, we finally included seven reports comprising 7,816 individuals for analysis (**Figure 1**) (Zhu et al., 2010; Yao et al., 2014; Riba-Llena et al., 2016; Ding et al., 2017; Liang and Chan, 2017; Banerjee et al., 2019; Park et al., 2019). Overall, they were cohort studies, with six reports published in a journal and only one was a conference abstract. Five of these studies were based in Europe, and two were based in Asia. Two reports were from the same study, but there was no conflict due to the different parts of ePVS studied (3C SG, 2003). Most papers used 1.5T MRI and T1 and T2 sequences to identify ePVS. Most studies reported on ePVS in the BG and CSO, although several papers reported ePVS in the HP and WM (Zhu et al., 2010; Yao et al., 2014). The detailed characteristics of all included studies are shown in **Table S1**.The quality of studies based on the NOS scores is presented in **Table 1**. All studies were of medium to high quality (score ≥ 7).

**Table 1:** Quality assessment of included studies.

References	Year	Selection	Comparability	Exposure	Total
(Park et al., 2019)	2019	★★★	★	★★★	7
(Banerjee et al., 2019)	2019	★★★	★	★★★	7
(Ding et al., 2017)	2017	★★★★	★★	★★	8
(Riba-Llena et al., 2016)	2016	★★★	★★	★★	7
(Yao et al., 2014)	2014	★★★	★★	★★★	8
(Zhu et al., 2010)	2010	★★★	★★	★★★	8
(Liang and Chan, 2017)	2017	★★	★★	★★★	7

### Associations Between ePVS and Cognitive Function

**Figure 2:**
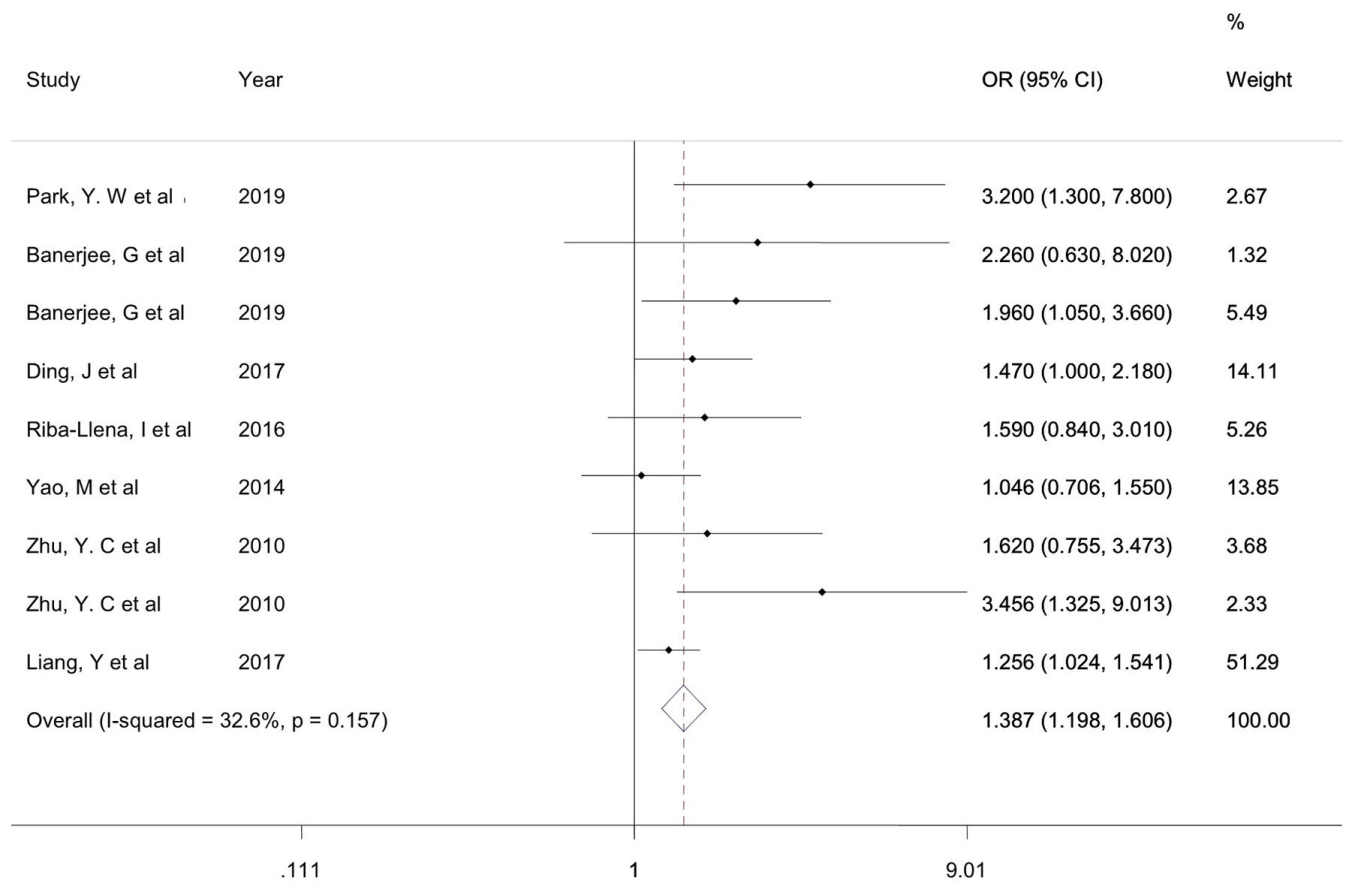
Forest plot of associations of ePVS and cognitive function. ePVS, enlarged perivascular spaces.

Seven studies with 7,816 individuals evaluated the association between ePVS and cognitive function. Heterogeneity test results indicated low heterogeneity (χ^2^ = 11.88, P=0.157, I^2^ = 32.6). The ePVS were significantly associated with cognitive function (OR = 1.387, 95% CI = 1.198–1.606, z=4.38, P<0.001) (**Figure 2**).

### Sensitivity Analysis and Publication Bias

**Figure 3:**
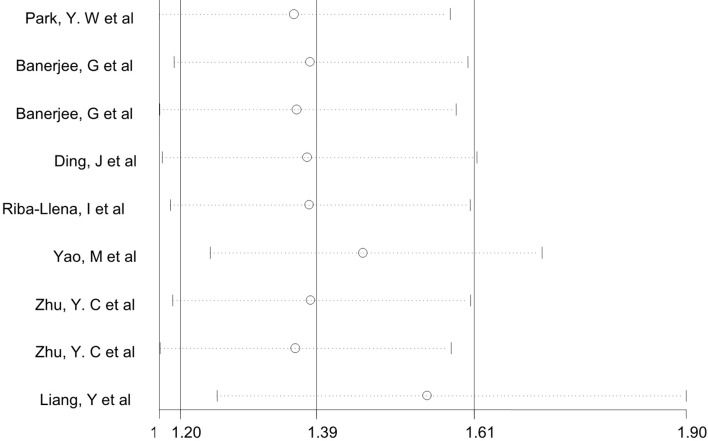
Sensitivity analysis used to assess the association between ePVS and cognitive function.

**Figure 4:**
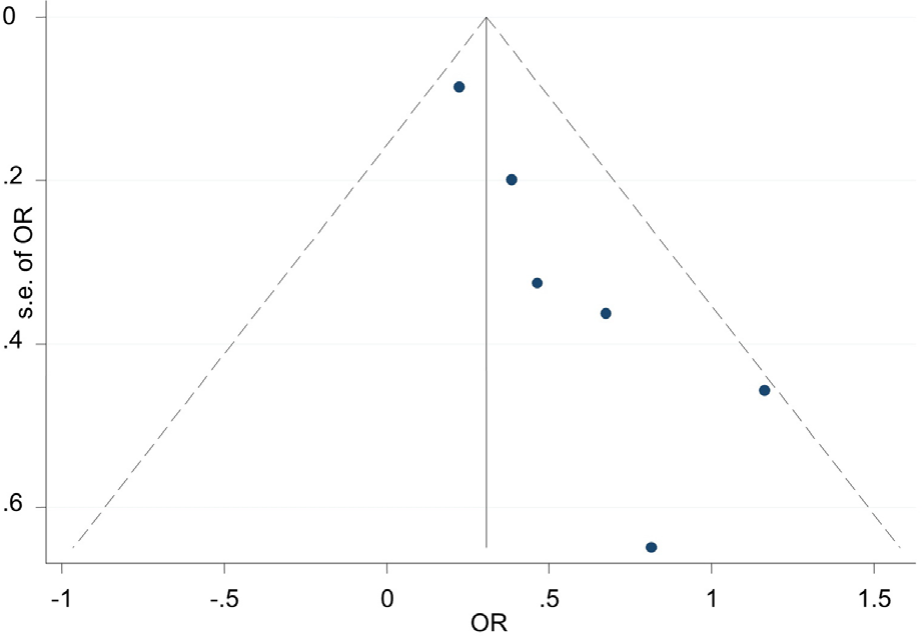
Publication bias was observed in the results based on Egger’s test (P = 0.009) and a funnel plot.

**Figure 5:**
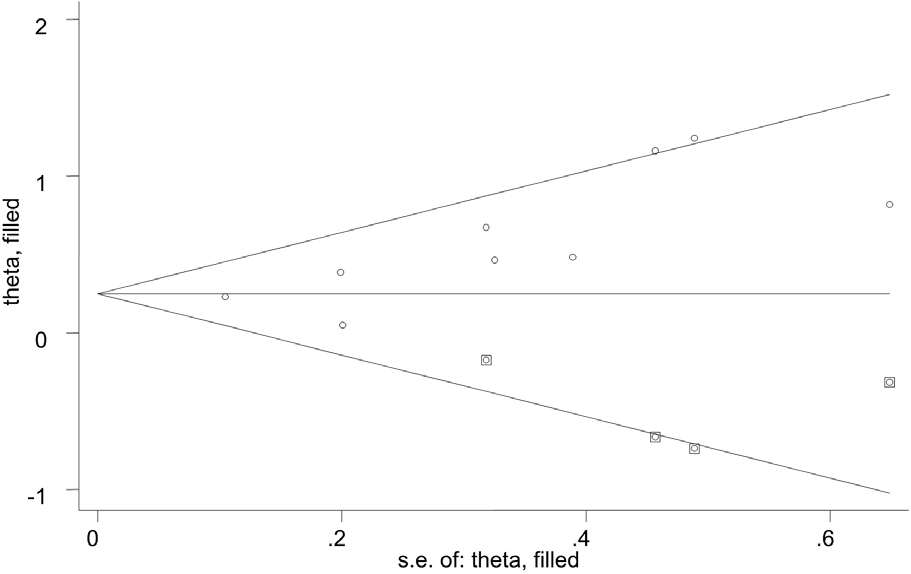
Trimming and supplementation was conducted to yield an effect adjusted for funnel plot asymmetry.

A sensitivity analysis was conducted by iteratively excluding individual studies from the analysis, and the results showed that no individual study influenced the overall OR (**Figure 3**), indicating that the results of this meta-analysis are relatively stable. Publication bias was observed in the results based on Egger’s test (P = 0.009) and a funnel plot (**Figure 4**). For the potential publication bias, we conducted a trim and fill analysis to yield an effect adjusted for funnel plot asymmetry. The result after trimming and supplementation (OR=1.297, 95% CI=1.130–1.490) was not significantly different from the previously determined OR (**Figure 5**).

## Discussion

This meta-analysis included seven studies involving a total of 7,816 participants. The combined analysis showed that ePVS counts were related to the risk of impaired cognition. To the best of our knowledge, this is the first meta-analysis in which the association between ePVS and risk of impaired cognition using longitudinal studies has been explored.

Previous studies, including cross-sectional studies (n=7), case-control studies (n=3), and cohort studies (n=5), have investigated the association of ePVS and cognition, but the results were inconsistent. Most of the cross-sectional studies using adjusted OR have suggested that ePVS were related to cognition, (Riba et al., 2015; Muir et al., 2016; Banerjee et al., 2017; Shams et al., 2017; Arba et al., 2018; Shibata et al., 2019) which was consistent with our findings. However, a study by Hurford et al. reported that ePVS do not have an independent association with cognitive impairment (Hurford et al., 2014). Three case-control studies using Spearman’s rank correlation coefficients all reported that increased ePVS counts may contribute to cognitive decline (Chen et al., 2011; Yun, 2013; Favaretto et al., 2017). With further review of the cohort studies, most of them have suggested that ePVS were related to cognitive decline (Van Westen et al., 2017; Jimenez-Balado et al., 2018; van Westen et al., 2018; Passiak et al., 2019). However, one small-sample cohort study reported that it was lacunes but not ePVS that were a predictor of cognitive decline (Trippier et al., 2018). In addition, a conference abstract based on a cohort study showed that hippocampal ePVS did not show any relation with MCI (Jimenez et al., 2017). A meta-analysis of population-based studies (Hilal et al., 2018) included only cross-sectional studies, which are more prone to recall bias, and these individuals may not have significant cognitive impairment, which may weaken the relationship between ePVS and cognitive dysfunction, which might explain the discrepancies between our findings and the findings of that study. However, surprisingly, there was no conclusive evidence that ePVS were related to specific clinical or neuroimaging features and a lack of specific meta-analysable data on ePVS and cognition.

In our study, the heterogeneity analysis indicated low heterogeneity, so we combined the adjusted ORs with the fixed effects model. However, to reduce the error caused by small samples, we compared the results of the random effects model and the fixed effects model and found that there was little difference between the two results. Despite the publication bias, we suggested that the publication bias had no significant effect on the results through the trim and fill method. The results were stable based on the sensitivity analysis. The subjects included in our meta-analysis included a variety of clinical diseases, such as Parkinson’s disease, Alzheimer’s disease and cerebrovascular disease, which have different clinical pathogenesis. However, these results were consistent with our meta-analysis. This implies that ePVS, previously considered one of the markers of small vascular diseases and was most closely related to vascular cognitive impairment (Li et al., 2018), may be a sign of cognitive impairment rather than a sign of particular diseases. We look forward to more studies to test this idea.

However, the article has some limitations. Some studies have suggested that different ePVS locations have different outcomes, and the etiology of ePVS in different locations may be different (Banerjee et al., 2017; Shams et al., 2017). Unfortunately, we were unable to conduct regional subgroup analyses due to the lack of sufficient data. Based on the available data, we could only conduct subgroup analysis in the BG, and the result (OR=1.360, 95% CI 1.178–1.570) was consistent with our conclusions. More data are needed to further analyze the relationship between the other three regions (BG, WM, and HP) and cognition. In addition, the methods used to evaluate ePVS in the studies we included were not completely consistent. Some studies (Maclullich et al., 2004; Trippier et al., 2018) have shown that ePVS and lacunes are sometimes indistinguishable, which may have influenced the results. In the future, studies should use consistent methods to determine a characteristic appearance of ePVS. In present, the potential of using automated methods to assess ePVS burden is being recognized. Boesp F et al (Boespflug et al., 2018) presented a fully automated method to extract enlarged PVS (ePVS) in clinical-field-strength MR imaging data. On this basis, Florian D et al (Dubost et al., 2019) improved the method and found that the PVS automated scoring method had good consistency and reproducibility which may replace visual scoring and facilitate large epidemiological and clinical studies of PVS.

## Conclusions

EPVS counts were associated with impaired cognition in risk-factor–adjusted meta-analysis. EPVS may be a sign of cognitive impairment rather than a sign a particular diseases. More research is needed to further validate ePVS as a measurable risk marker for cognition using consistent methods to determine a characteristic appearance of ePVS.

## Data Availability Statement

The raw data supporting the conclusions of this article will be made available by the authors, without undue reservation, to any qualified researcher.

## Author Contributions

HZ and ZL contributed to the conception and design of the meta-analysis. WJ, GLin, LL, and GLia were involved in the acquisition and analysis of the data. MO and ML interpreted the results. WJ and GLin drafted the manuscript. All authors read and approved the final manuscript.

## Funding

This work was supported by funding from the Natural Science Foundation of China (grant number 81400986), the Science and Technology Planning Project of Zhanjiang (grant number 2018A01021) and the Affiliated Hospital of GDMU (BJ201515, BJ201612 and LCYJ2018A003).

## Conflict of Interest

The authors declare that the research was conducted in the absence of any commercial or financial relationships that could be construed as a potential conflict of interest.
