# Supplementary material for: The Relationship Between Enlarged Perivascular Spaces and Cognitive Function: A Meta-Analysis of Observational Studies
Source: Front Pharmacol. 2020 May 15;11:715. doi: 10.3389/fphar.2020.00715 (PMC7243265; doi:10.3389/fphar.2020.00715)
Supplement: Supplementary file 1 [file Table_1.docx]

| No. | References | Publication Year | Country of publication | Publication type | Sample source | Sample size | Age (mean) | Subject characteristics |
| --- | --- | --- | --- | --- | --- | --- | --- | --- |
| 1. | Park, Y. W et al.^8^ | 2019 | United States | Journal | Yonsei Parkinson Center database(Asia) | 271 | NA | PD |
| 2. | Banerjee, G et al.^90^ | 2019 | Germany | Journal | The CROMIS-2 AF Study  (Europe) | 114 | 73.1 | Ischaemic Cardioembolic Stroke or TIA with Non-valvular Atrial Fibrillation |
| 3. | Ding, J et al.^9^ | 2017 | United States | Journal | AGES-Reykjavik Study(Europe) | 2612 | 74.6 | population-based study of older persons |
| 4. | Riba-Llena, I et al.^15^ | 2016 | England | Journal | the ISSYS project(Europe) | 798 | 62.8 | essential hypertensive individuals |
| 5. | Yao, M et al.^7^ | 2014 | United States | Journal | 3C-Dijon MRI Study(Europe) | 1745 | 72.46 | population-based study of older persons |
| 6. | Zhu, Y. C et al.^6^ | 2010 | Netherlands | Journal | 3C-DijonMRI Study(Europe) | 1778 | 72.4 | population-based study of older persons |
| 7. | Liang, Y et al.^2^ | 2017 | Netherlands | Conference Abstract | Prince Wales Hospital of Hong Kong(Asia) | 498 | 65.8 | The first-ever acute ischemic stroke patients |

| No. | Follow-up time | Grouping | ePVS Location | result, OR/RR/HR(95%CI) | adjustment factors | Statistical method | EPVS Rating Scale | Type of scanner MRI sequence | Psychological assessment method |
| --- | --- | --- | --- | --- | --- | --- | --- | --- | --- |
| 1. | 59.8months | ALL | BG | 3.2(1.3-7.8) | Sex | Logistic regression analyses | 4 points | 3T,T2 and FLAIR | MMSE/BDI/CCSIT |
| 2. | 12months | non reverters | BG  CSO | 2.26 (0.63–8.02)  1.96 (1.05–3.66) | Acute MoCA score | Logistic regression analyses | 4 points | NA,T2 and FLAIR | MOCA |
| 3. | 62.4months | All-cause dementia | BG | 1.32(0.89-1.97) | age, sex, interval between the baseline, follow-up MRIs, head coil,body mass index,current smoking,hypertension,total cholesterol level,prevalent symptomatic | Logistic regression analyses | 3 points | 1.5T,T1,T2,GRE,FLAIR | MMSE/DSST/TMT A and B / RAVLT |
| 4. | 36 months | MCI | BG | 1.59( 0.84-3.01) | Education, sex, high grade deep WMHs and lacunes,  diabetes mellitus, age, BPLD compliance, microalbuminuria, | Logistic regression analyses | 4 points | 1.5T，T1,T2,FLAIR | DRS-2/RAVLT/ WMS-III/TMT A and B / COWAT /Stroop test /Clock drawing/ Barcelona's test |
| 5. | 73.4 months | Degree 1  Degree 2 | HP  HP | 1.13 (0.70-1.84)  0.90 (0.46-1.78) | age, gender and educational level,apoE4 genotype, risk number of cerebral vascular disease, and the presence of depressive symptoms, brain parenchymal fraction, normalized volume of white matter hyperintensities, and the presence of lacunar infarcts | Logistic regression analyses | 3 points | 1.5T，T1,T2,3D IR-SPGR | MMSE/IST/TMT A and B/BVRT |
| 6. | 48 months | Degree 2  Degree 3  Degree 4 | BG  WM  BG  WM  BG  WM | 1.7 (0.7–4.6)  3.1 (0.7–13.9)  0.7 (0.1–3.6)  1.7 (0.3–10.4)  3.4 (0.5–22.1)  8.1 (1.4–47.5) | age, apolipoprotein E4, and total intracranial volume, WMH volume and presence of infarct | Logistic regression analyses | 4 points | 1.5T，T1,T2,3D IR-SPGR | MMSE/IST/TMT A and B |
| 7. | 15months | 15 months | BG | 1.256(1.024-1.541) | age, sex, education, NIHSS score, volume of acute infarct, and other markers of CSVD | Logistic regression analyses | 4 points | NA,T1,T2 | MMSE |

Table S1. Characteristics of studies included in the meta-analysis
